# Intranasal therapies for neonatal hypoxic-ischemic encephalopathy

**DOI:** 10.4103/NRR.NRR-D-25-01036

**Published:** 2025-12-30

**Authors:** Andrew S. Cavanagh, Benjamin I. Sollinger, Nazli Kuter, Khyzer Aziz, Victoria Turnbill, Lee J. Martin, Frances J. Northington

**Affiliations:** 1 Department of Pediatrics, Division of Neonatology, Johns Hopkins School of Medicine, Baltimore, MD, USA; 2 Undergraduate Programs in Neuroscience and Molecular and Cellular Biology, Johns Hopkins Krieger School of Arts and Sciences, Baltimore, MD, USA; 3 Department of Neurology and Developmental Medicine, Kennedy Krieger Institute, Baltimore, MD, USA; 4 Departments of Pathology, Neuroscience, and Anesthesiology & Critical Care Medicine, Johns Hopkins School of Medicine, Baltimore MD, USA; 5 Pathobiology Graduate Training Program, Johns Hopkins School of Medicine, Baltimore, MD. USA

**Keywords:** anti-inflammatory drugs, cell therapies, cellular stress, drug development, global health, growth factors, hypoxia-ischemia, intranasal therapies, neonatal brain injury, systematic review

## Abstract

Neonatal hypoxic-ischemic encephalopathy is the leading cause of brain injury in term infants worldwide and disproportionately affects low- and middle-income communities. Therapeutic hypothermia, the standard of care for hypoxic-ischemic encephalopathy in high-resourced settings, has no effect on morbidity and increases mortality after hypoxic-ischemic encephalopathy in low- and middle-income settings. Intranasal administration offers the opportunity to deliver more accessible treatments for all babies with neonatal hypoxic-ischemic encephalopathy due to lower resource needs, ease of administration, and the capacity to directly target the brain. We reviewed preclinical literature concerning intranasal treatments for hypoxic-ischemic encephalopathy and developed a novel semi-quantitative index ranking intranasal therapies for their potential for further development as biologically plausible, effective, and accessible treatments for hypoxic-ischemic encephalopathy. We searched PubMed and Google Scholar for peer-reviewed articles on intranasal therapies for hypoxic-ischemic encephalopathy using the mesh phrases “Neonatal hypoxic-ischemic encephalopathy intranasal” and “Neonatal brain intranasal.” Sixty-two studies were included that described thirty-four unique intranasal therapies. Neonatal intranasal therapies have been widely studied in small animal models, infrequently in large animals, and only recently in human clinical trials. Our semi-quantitative ranking revealed cell-based therapies as potentially the most effective and developed intranasal therapy in animal models of hypoxic-ischemic encephalopathy, though the pharmaceutical support compulsory to current cell-based treatments limits their accessibility in low-resourced settings. Intranasal therapies for neonatal hypoxic-ischemic encephalopathy have both feasibility and neuroprotective potential for safe, effective, and accessible treatment of hypoxic-ischemic encephalopathy. Additional research is needed for translation to humans. Future investigation should emphasize appropriate animal modeling with pharmaceutics and cells, combined with an evaluation of the brain connectome and neurobehavioral outcomes.

## Background

Neonatal hypoxic-ischemic (HI) encephalopathy (HIE) is the leading cause of neurodevelopmental morbidity in term infants (Kurinczuk et al., 2010). While the 2019 incidence of neonatal encephalopathy was as low as 6.51/100,000 population in high-income countries, the 2019 incidence was as high as 19.75/100,000 population in South Asia and 53.59/100,000 population in Sub-Saharan Africa (Ji et al., 2025). South-Southeast Asia and Sub-Saharan Africa contribute to an estimated 85% of global HIE cases (Lee et al., 2013; Ji et al., 2025). The current standard of care for HIE is therapeutic hypothermia (TH), which decreases morbidity and mortality in high-income communities (Shankaran et al., 2005). In contrast, the HELIX trial showed no effect on morbidity and increased mortality with TH in low-middle income communities (LMICs) (Thayyil et al., 2021). A lack of access to specialized neonatal care is not confined to LMICs. As of 2013, only 50% of women living in non-metropolitan labor-market, or micropolitan, areas, and less than 30% of women living in rural areas in the United States lived within a 30-mile drive of a neonatal intensive care unit (Hung et al., 2018). Accessibility to therapeutics for infants born in all communities needs prioritization in the search for novel therapies for HIE.

Intranasal (IN) administration of neurotherapeutics has emerged because of its minimally invasive nature and high central nervous system bioavailability (Pires et al., 2009). IN therapies have been tested clinically for over four decades, starting with Parkinson’s disease, Alzheimer’s disease, and myasthenia gravis (Jensen, 1980; Chung et al., 2023). These pioneering studies established that IN administration of drugs can be clinically efficacious with ease of application and minimal discomfort and stress in adult patients. IN medications such as analgesics and sedatives are now often delivered to pediatric patients, including neonates (Guastella et al., 2010; Warrington and Kuhn, 2011; Perri et al., 2025). Children and adolescents with autism are also treated with IN therapeutics (Guastella et al., 2010; Sikich et al., 2021). We propose that IN therapeutics could be adopted for treating infants with HIE. We review basic differences in rodent and human olfactory anatomy and then review the literature on the use of intranasal therapeutics for the treatment of hypoxia-ischemia in neonatal animal models. We conclude by proposing a novel semi-quantitative method for comparing the development of experimental IN therapeutics for HIE.

### Mechanisms of intranasal therapeutic delivery to the brain

**Figure 1:**
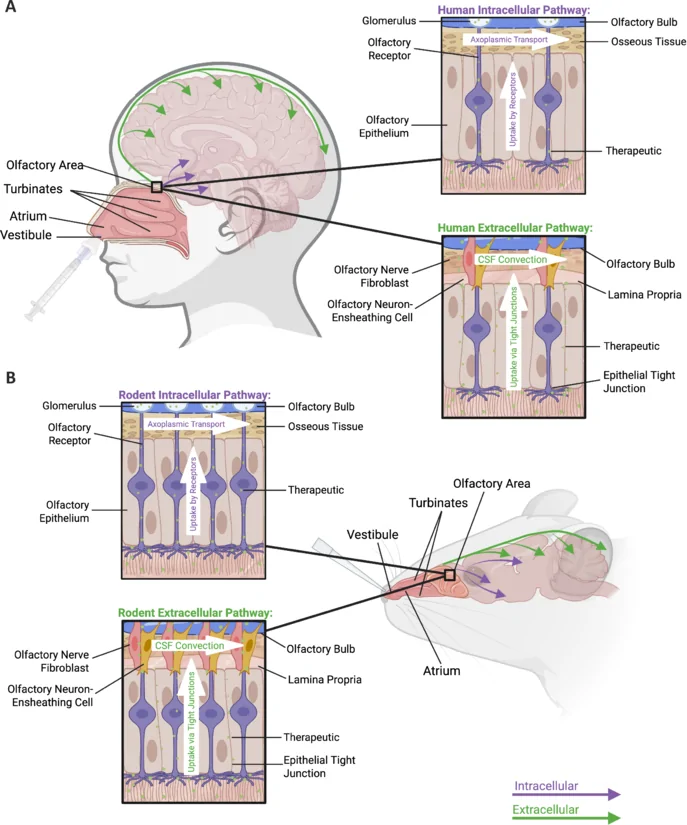
Human and mouse nasal anatomy and histology. Shown are anatomic and cellular structures of the human (A) and mouse (B) nasal cavity and olfactory epithelium relevant to the intranasal delivery of therapeutics to the central nervous system. Olfactory sensory neurons (purple cells), olfactory nerve fibroblasts, and olfactory neuron ensheathing cells (red and yellow cells) are portrayed. Created with BioRender.com. CSF: Cerebrospinal fluid.

IN therapeutic delivery begins with administration of liquid drugs, liposomes, exosomes, or intact cells directly into the nostrils as a nasal spray or spritz. Therapeutics then access the brain by traversing a highly elegant olfactory anatomy (Crowe et al., 2018; **Figure 1**), or IN therapeutics access the brain through the trigeminal nerves and adjacent pathways (Ganger and Schindowski, 2018). Multiple structures need to be considered, including the olfactory mucosa, the submucosa, the peripheral nerve supply, the arterial/venous supply, and lymphatics (Gizurarson, 2012). Access to the brain begins at the epithelial mucosa with its respiratory epithelial cells, Bowman’s glands, olfactory epithelium (bipolar olfactory receptor sensory neurons), and basement membrane (Han et al., 2023). The axons of the olfactory bipolar neurons, forming the olfactory nerve layer, pass through the cribriform plate of the ethmoid bone and synapse on the mitral cell and tufted cell dendrites in the glomerular layer of the olfactory bulb. The human olfactory bulb is about 12 mm in length (Liu and Martin, 2003). The olfactory tract, containing axons from the mitral cells, issues from the olfactory bulb and has a length of about 21 mm in humans (Lang and Reiter, 1984). The axons synapse in the piriform cortex (primary olfactory cortex), amygdala, and entorhinal cortex. Though this anatomy is generally conserved among species, there are many key differences between the rodent and human nasal mucosa, olfactory epithelium, olfactory bulbs, and brain targets of the olfactory cranial nerve (Kikuta et al., 2024). The olfactory epithelium is uniform and cleft-like in humans, while it is recessed and separated from the nasopharynx in rodents. The olfactory epithelium is proportionally larger in rodents, with a higher density of mature olfactory sensory neurons compared to humans (**Figure 1**).

Exogenous IN therapeutics can access the brain by both intracellular and extracellular routes (Crowe et al., 2018; **Figure 1**). The intracellular route includes endo- and pinocytosis by olfactory receptor neurons, axoplasmic trafficking in olfactory or trigeminal nerve axons, exocytosis of the therapeutic, and distribution throughout the CNS (Kristensson and Olsson, 1971; Jansson and Bjork, 2002). The axonal transport can be anterograde from the olfactory sensory neurons to the olfactory bulb. Extracellular distribution of a therapeutic to the brain includes crossing of olfactory epithelial tight junctions into paracellular clefts, diffusion through perineural space between olfactory neuron-ensheathing cells and olfactory nerve fibroblasts, access to subarachnoid space, and delivery to CNS tissue via cerebrospinal fluid convection. Systemic transport via blood and lymphatic vasculature through olfactory epithelium may also deliver IN therapeutics to the CNS (Kida et al., 1993; Johnston et al., 2004). Once drugs access the extracellular milieu of the olfactory bulb, they could be retrogradely transported to the nucleus of the diagonal band of Broca (Senut et al., 1989). Axoplasmic transport of some substances via the olfactory tract takes hours, but solute transport through the perineuronal space is often experimentally much faster (Broadwell and Balin, 1985). While these results may indicate extracellular routes as the primary means of drug delivery to the brain, the relative contributions of intracellular and extracellular therapeutic trafficking to the brain are complex and variable depending on intracellular transport type (anterograde or retrograde) and therapeutic characteristics, including size, solubility, and charge (Thorne and Frey, 2001; Crowe et al., 2018).

Neonatal-adult pharmacokinetic differences for IN neurotherapeutics are relatively unknown. It is also important to determine if olfactory neurons or neuroepithelium are damaged by HIE and if injury alters IN drug delivery to the CNS (Moreira et al., 2005; Gizurarson, 2012). The olfactory mucosa could be damaged by hypoxia and ischemia (Nakashima et al., 1985; Kim et al., 2023), as the olfactory system is damaged in patients after cardiac arrest (Aksit and Cil, 2020). The piriform cortex appears to become hypermetabolic and is damaged in HIE piglets (Martin et al., 1997; Wang et al., 2015). Thus, IN delivery of experimental therapeutics for HI demands proof of brain penetrance in control and injury conditions (Huang et al., 2023).

### Optimizing the accessibility of therapeutics for hypoxic-ischemic encephalopathy

Despite the excessive HIE burden in LMICs, many issues impede the widespread implementation of various therapies for HIE. Effective TH must be initiated within the first 6 hours of birth for the treatment of HIE (Shankaran et al., 2005), and many communities lack adequate prehospital transportation infrastructure to meet that time constraint (Suryanto et al., 2017). Treatments that are therapeutic when administered well beyond the first 6 hours after birth are urgently needed, regardless of administration route. Other extrinsic and intrinsic factors impacting the ability to deliver a therapy include cost of the therapy and distribution, need for refrigeration, solubility, ease and route of administration, dosing regimen, and follow-up care (Jalali et al., 2022). IN therapies may be more easily administered and repeatedly dosed, if necessary, than other currently available treatments for HIE (Vyas et al., 2005; Pires et al., 2009; McDermott, 2012; Inokuchi, 2015). They may also pose less risk for adverse complications compared to IV (intravenous) administration, and non-cellular IN therapies can be delivered outside of structured neonatal units by non-medically trained, yet competent, personnel if extended dosing is needed (Ben Abdelaziz et al., 2017).

### Safety concerns

The IN administration of analgesics and sedatives is approved for human neonates, suggesting IN drug delivery to the neonatal nervous system can be safe and effective (Ku et al., 2019; Kaushal et al., 2020; Snyers et al., 2022), at least in limited use or single administration. Physical and chemical irritation of the nasal and nasopharyngeal epithelium is a concern with IN administration (Gizurarson, 2012). Coadministration of lidocaine has been used for this purpose (Linakis et al., 2016), but lidocaine exposure, and other agents administered to decrease irritation, may cause untoward side-effects, including seizures (Resar and Helfaer, 1998), limiting the enthusiasm for IN treatments that are irritating or painful. As neonates are obligate nose breathers, the volume of IN agents must be closely monitored to avoid respiratory compromise and aspiration (Derkay and Grundfast, 1990). Administration of large volumes of human breastmilk to preterm infants for intraventricular hemorrhage has been reported without significant safety concerns (Keller et al., 2019; Hoban et al., 2024; Sonmez Demir et al., 2025). Therapies can be administered as IN pulses rather than large-volume boluses to prevent aspiration (Derkay and Grundfast, 1990).

## Methods

### Search strategy

We followed the Preferred Reporting Items for Systematic Reviews and Meta-Analyses (PRISMA) 2020 guidelines in literature search and construction of this review (Page et al., 2021). We compiled literature with mesh terms, “Neonatal Hypoxic-Ischemic Encephalopathy Intranasal” and “Neonatal Brain Intranasal,” on PubMed and Google Scholar search engines as of November 16, 2024. 103 peer-reviewed articles were evaluated for inclusion. Three authors reviewed each article for the inclusion criteria. Literature was included if it presented original, peer-reviewed research concerning the IN administration of an experimental therapeutic for HIE published in a journal with an impact factor greater than 2. Peer-reviewed articles utilizing infection-sensitized models of HI and models of preterm HI brain injury were also included. Articles not meeting the inclusion criteria were excluded. Article evaluation for each mesh term on each search engine was ceased after 10 consecutive articles were excluded. Additionally, human clinical trials of IN mesenchymal stromal cells (MSC) for perinatal arterial ischemic stroke (PAIS) and IV MSCs for HIE were included as the first of their kind for human neonatal brain injury treatments (Baak et al., 2022; Cotten et al., 2023).

### Semi-quantitative index

**Figure 2:**
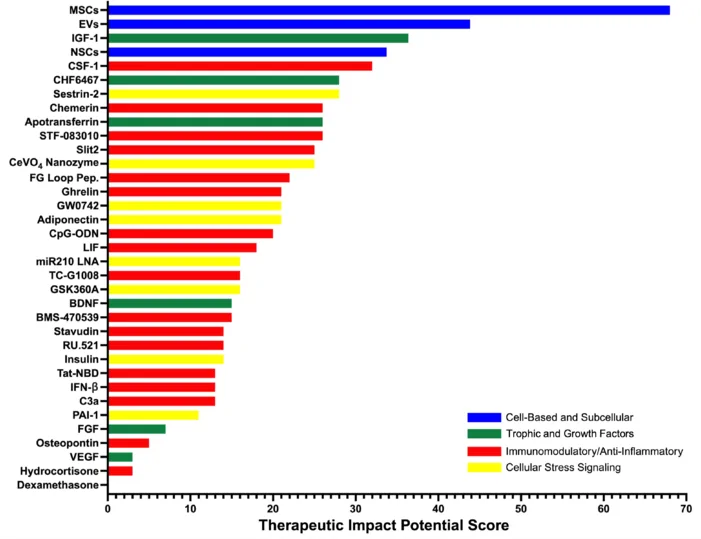
Therapeutic impact potential. Therapies are indexed according to the method described in the Methods. Therapies similar in mechanism are color-coded according to the legend on the right. Created with BioRender.com. BDNF: Brain-derived neurotrophic factor; CSF-1: colony-stimulating factor 1; EVs: extracellular vesicles; FGF: fibroblast growth factor; IFN-β: interferon beta; IGF-1: insulin-like growth factor 1; LIF: leukemia inhibitory factor; MSCs: mesenchymal stromal cells; NSCs: neural stem cells; ODN: oligodeoxynucleotide; VEGF: vascular endothelial growth factor.

We created a semi-quantitative index of relative therapeutic development for IN therapies. Our index, termed the therapeutic impact potential (TIP), quantified the efficacy and developmental progress of IN therapeutics towards demonstrating a positive result on common experimental outcomes. A point-based scoring system was devised to compare therapies. Original research articles were reviewed by two authors, and experimental outcomes were compiled. Each therapeutic was assigned a score of 1 for each outcome if a positive result was demonstrated (*P* < 0.05) or 0 if a negative result was demonstrated or if the outcome was not measured. Each repetitive demonstration of a positive outcome across independent laboratories was assigned a compounding half-point value. For example, if a positive outcome was replicated across three independent studies conducted in independent laboratories for a given therapy, that therapy was designated 1.75 points: 1 point for the first report, 0.5 points for the second report, and 0.25 points for the third report. Individual experimental outcomes were grouped into broader classifications as shown in **Figure 2**. Points were summed within classifications and multiplied by a weighting factor unique to the classification. The authors determined outcome classifications and weighting factors based on their importance to accelerating further testing of therapies. Classifications and weightings were as follows: demonstrated therapeutic brain penetrance with IN dosing (weighting=5), positive neurobehavioral outcome (weighting=4), improved histopathology (weighting=3), reduced inflammation (weighting=2), and evidence for regulating a specific molecular mechanism known to affect the HI-injured brain (weighting=1). When brain penetrance was not considered in the scoring, respective weighting factors for all other outcome classifications remained. Weighted scores for all outcome classifications were summed to determine a total TIP score for each therapy (**Figure 2**).

## Discussion

Within the 62 included articles published from 2009 to 2024, 34 unique IN therapies were presented. Details of injury model, dose, dosing regimen, and experimental results for all studies reviewed are presented in **Additional Table 1**. Therapies are presented within each subsection in order of publication date.

18 of 34 therapies have explicitly demonstrated brain penetrance with IN dosing by quantitative imaging or post-vivo methods. The half-lives of IN pharmaceuticals must be considered in pharmacokinetic studies. Mucociliary clearance, enzymatic degradation, lipophilicity-hydrophilicity balance, and inherent stability may all affect the half-life and absorption of IN therapeutics (Thorne and Frey, 2001). Half-lives and bioavailability of these compounds may also be affected by interactions with binding proteins and components of the extracellular matrix (Thorne and Frey, 2001). These factors are the basis for recommending pharmacokinetic/pharmacodynamic studies in each new model tested for an IN therapy.

### Cell-based and subcellular therapies

#### Mesenchymal stromal cells

MSCs are the most studied IN therapy for HIE. However, MSCs administered by any route have not advanced to phase 3 clinical trial for HIE (Cotten et al., 2023). In a pilot phase I trial of 1–2 doses of IV human (h) umbilical cord blood–derived MSCs (2 × 10^6^ cells/kg) as an adjunct to TH for HIE, hMSCs were well-tolerated. Importantly, 5/6 babies developed anti-HLA antibodies by 1 year. All survived with average to low-average neurodevelopmental assessment at 12–17 months (Cotten et al., 2023). A phase I trial has evaluated IN allogenic bone marrow-derived hMSCs for PAIS in the middle cerebral artery region (Baak et al., 2022). 4.5–5 × 10^5^ IN MSCs were well tolerated in ten neonates, > 36 weeks’ gestation. No adverse events requiring clinical intervention occurred. There were no changes in blood inflammation markers after MSC administration (Baak et al., 2022). Though this trial in neonates was with PAIS, rather than HIE, it supports the safe IN administration of hMSCs for neonatal brain injury. Future studies, including continued clinical study of IN MSCs for PAIS, are important to support the feasibility of IN cell therapies for neonatal HIE. Confirmation of therapeutic MSC brain penetrance is needed. Cell tracking using non-invasive imaging methods, including contrast particle-labeled stem cells, could prove the bioavailability of IN cell therapies in the brains of human neonates (Huang et al., 2023). Sustained genomic stability of exogenous cells needs to be studied rigorously to avoid oncogenic transformation. Parenchymal dispersal of cells needs to be shown because of the fear of exogenous isochronic cell affinity to form intracerebral masses. Discussion of patient/subject immunosuppression for use of allogenic hMSCs in the PAIS and other human trials is also needed.

Studies investigating IN MSCs in animal models of HIE are few. IN MSCs are brain penetrant, as measured *in-vivo* using superparamagnetic iron oxide particle-labeled MSCs and post-vivo using PKH-26 red fluorescent-labeled MSCs, in several rodent and piglet models (van Velthoven et al., 2010, 2014; Donega et al., 2013, 2014a, b, 2015; Herz et al., 2018; McDonald et al., 2019; Robertson et al., 2021; Jiao et al., 2022; Hermans et al., 2024). IN MSCs consistently reduce HI infarct size and resolve motor and cognitive deficits (van Velthoven et al., 2010, 2014; Donega et al., 2013, 2014a, b, 2015; Herz et al., 2018; McDonald et al., 2019; Robertson et al., 2021; Jiao et al., 2022; Hermans et al., 2024). IN MSCs reach the neocortex and hippocampus with higher abundance near the lesion site. The therapeutic window for IN MSCs in rodent models is 10–17 days post-HI, which greatly exceeds the therapeutic window for TH, improving the potential accessibility for treatment of HIE (van Velthoven et al., 2010, 2014; Donega et al., 2013, 2014a, b, 2015; Hermans et al., 2024). MSC treatment upregulates several growth factors and downregulates pro-inflammatory cytokines with resolution of glial activation and scarring (van Velthoven et al., 2010; Donega et al., 2014a, b; Herz et al., 2018; McDonald et al., 2019; Jiao et al., 2022). IN human amniotic fluid stem cells and amniotic epithelial cells are therapeutic after HI in rodents when given at 10 days or 1-, 3-, and 10-days post-injury, respectively (Otani et al., 2019; van den Heuij et al., 2019). In fetal sheep with HI, IN human amniotic fluid stem cells protect against neuron and oligodendrocyte damage and attenuate inflammatory changes seen at 21 days post-injury, though there was no tracking of human cells in the sheep brain (van den Heuij et al., 2019). These findings are astonishing given that there was no immunosuppression to prevent graft-*versus*-host rejection. In previous preliminary studies of autologous olfactory bulb pig neuroprogenitor cell transplantation into neonatal HI piglets, a strong host inflammatory cell response was seen (Martin, 2009).

Several factors improve the delivery and efficacy of IN MSCs, including expression of brain-derived neurotrophic factor, EGFL7, or cytoglobin and administration of chemoattractant, and CXCL10 (Donega et al., 2014b; van Velthoven et al., 2014; Yang et al., 2020; Hermans et al., 2024). IN hMSCs are therapeutic in a murine model of infection-sensitized HI injury, in contrast to TH (Jiao et al., 2022). The safety and efficacy of IN MSCs as an adjunct to TH is unclear, as mouse MSCs with TH worsen behavioral deficit, infarct size, and immune cell infiltration compared to each treatment alone in a murine model. In contrast, IN hMSCs are therapeutic combined with TH in a piglet model (Robertson et al., 2021), and IV hMSCs as an adjunct to TH are reported as safe in 6 human patients (Cotten et al., 2023).

#### Extracellular vesicles

Extracellular vesicles (EV) are derived from various cell sources and deliver a diverse array of biomolecular cargo, including miRNAs, proteins, lipids, and nucleic acids. IN EVs derived from human and mouse neural stem cells (NSCs), human and mouse brain, primary MSCs, and clonally immortalized expanded MSCs are brain penetrant and reduce gray and white matter atrophy after HI in rodents (Lawson et al., 2022; Turovsky et al., 2022; Labusek et al., 2023a, b; Tscherrig et al., 2024). Several studies show resolution of cognitive and sensorimotor impairment with IN EVs. IN EVs decrease glial and endothelial cell activation, improve oligodendrocyte maturation, and decrease apoptosome activation after HI. EVs contain miRNAs, including some downregulated by HI such as miR-342-3p and miR-330-3p, as well as several proteins. Despite the disproportionately high abundance of proteins in EVs compared to nucleic acids, DROSHA nuclease knockdown in MSCs producing EVs leads to diminished EV therapeutic efficacy, indicating a role for miRNAs in the therapeutic effects of IN EVs (Lawson et al., 2022; Turovsky et al., 2022; Labusek et al., 2023a, b; Tscherrig et al., 2024).

#### Neural stem cells

IN human NSCs reduce gray and white matter injury and improve cognitive and sensorimotor behavior in rodents after HI (Ji et al., 2015; Ye et al., 2018b; Lu et al., 2022). NSCs overexpressing basic fibroblast growth factor differentiate into more neurons and are more effective at resolving somatosensory impairment in mice than NSCs with normal basic fibroblast growth factor expression (Ye et al., 2018b). Pro-inflammatory cytokine expression is reduced with IN hNSCs (Ji et al., 2015). Hypoxic pretreatment, delivery with an IN catheter, and repeated dosing at intervals greater than 24 hours improve delivery of IN hNSCs to the brain (Lu et al., 2022).

### Trophic and growth factors

#### Insulin-like growth factor 1

IN human recombinant insulin-like growth factor 1 (IGF-1) is brain penetrant, reduces apoptotic death through the Akt signal transduction pathway, enhances proliferation of neuronal and oligodendroglial progenitors, and improves performance on a passive avoidance task after neonatal HI in rats (Lin et al., 2009). TH extends the therapeutic window of IN human recombinant IGF-1 from 2 to 6 hours post-HI accompanied by a decrease in polymorphonuclear leukocyte infiltration, microglia/macrophage activation, and nuclear factor-κB (NF-κB) activation (Lin et al., 2011).

#### Apotransferrin

Apotransferrin is brain penetrant, improves survival and maturation of oligodendroglia, improves myelination, reduces neuronal loss and astrogliosis, increases proliferation of neurons and oligodendroglia, and reduces caspase 3 expression when delivered IN after neonatal HI in mice (Guardia Clausi et al., 2012).

#### Fibroblast growth factor

IN acidic fibroblast growth factor or basic fibroblast growth factor reduces infarct volume, cleaved caspase 3 expression, and neuron cell death after neonatal HI in rats (Hu et al., 2017; Lin et al., 2017). Both reduce expression of endoplasmic reticulum stress-related proteins (Hu et al., 2017; Lin et al., 2017).

#### Vascular endothelial growth factor

IN human recombinant vascular endothelial growth factor promotes recovery of cerebrovascular density after neonatal HI in piglets without promoting neovascularization (Jain et al., 2017).

#### Modified nerve growth factor

CHF6467, a recombinant form of human nerve growth factor devoid of allogenic activity, reduces infarct size after neonatal HI in rats (Landucci et al., 2024) and is synergistic with TH. IN CHF6467 reduces mRNA upregulation of inflammatory cytokines and an increase in plasma levels of neurofilament light chain. In hippocampal slices exposed to oxygen and glucose deprivation, CHF6467 reduces cell death and synaptic transmission impairment (Landucci et al., 2024).

#### Brain-derived neurotrophic factor

IN brain-derived neurotrophic factor is brain penetrant, increases synaptophysin and MAP2 expression, and improves performance on a novel object recognition task after neonatal HI in mice (Sims et al., 2024).

### Cellular stress signaling

#### Plasminogen activator inhibitor-1

IN CPAI-1, a stable mutant form of PAI-1, is brain penetrant, decreases infarct size, and diminishes activity of both tissue and urinary-type plasminogen activators and matrix metalloproteinase-9 after neonatal HI in mice (Yang et al., 2013b). IN CPAI-1 inhibits NF-κB activation, blocks the increase in interleukin-6 and Ccl-2, and reduces microgliosis in an lipopolysaccharide-sensitized mouse model (Yang et al., 2013b).

#### Sestrin2

IN recombinant human Sestrin2 is brain penetrant, reduces infarct size, resolves cognitive and sensorimotor impairment, and stabilizes BBB permeability after neonatal HI in mice (Shi et al., 2016, 2017). Human recombinant-sestrin2 increases AMP-activated protein kinase phosphorylation, inhibits mammalian target of rapamycin, and increases junction protein expression (Shi et al., 2016, 2017).

#### miR-210 complementary locked nucleic acid

Inhibition of miR-210 by IN administration of its complementary locked nucleic acid is brain penetrant, reduces infarct size and cognitive and motor deficits after neonatal HI in rodents (Ma et al., 2016).

#### Adiponectin

IN adiponectin is brain penetrant, decreases infarct size and neuronal death, and relieves cognitive and sensorimotor deficits after neonatal HI in rodents (Xu et al., 2018).

#### PPAR-b/d agonist

IN administration of GW0742, a PPAR-b/d agonist after neonatal HI in rats reduces infarct size and relieves cognitive and sensorimotor impairments (Gamdzyk et al., 2018).

#### Hypoxia inducible factor stabilization

GSK360A, a potent prolyl-4-hydroxylase inhibitor, is brain penetrant and reduces cortical neuroinflammatory markers and infarct area when given intracerebroventricularly or IN, but not via intraperitoneal injection after neonatal HI in rodents (Kuan et al., 2021). IN GSK360A triggers upregulation of erythropoietin, heme oxygenase 1, and glucose transporter 1 transcripts (Kuan et al., 2021).

#### Insulin

IN insulin after neonatal HI in rodents is brain penetrant, reduces infarct size and neuronal death, increases Akt phosphorylation, and diminishes short-term sensorimotor dysfunction (Talati et al., 2024).

#### Cerium vanadate nanoenzyme

A cerium vanadate (CeVO_4_) nanoenzyme penetrates the brain, colocalizes with neuronal mitochondria, and reduces neuron cell death and gliosis when delivered IN after neonatal HI in mice (Jiang et al., 2024). IN CeVO_4_ also reduces reactive oxygen species, restores nuclear factor erythroid 2-related factor 2 expression, increases cortical dendritic spine density, and reduces infarct size and neurobehavioral deficits (Jiang et al., 2024).

### Immunomodulatory and anti-inflammatory agents

#### Anti-nuclear factor-κB peptides

IN administration of Tat-NBD, a peptide-inhibitor of NF-κB activation and nuclear translocation was found to penetrate the brain, and reduce cortical infarct size, NF-κB activation, microglial activation, and neuronal apoptosis in a rodent model of lipopolysaccharide exposure-sensitized neonatal HI (Yang et al., 2013a).

#### Osteopontin and osteopontin-derived peptides

Despite evidence of therapeutic brain penetrance, neither mouse recombinant osteopontin nor TAT-osteopontin peptides relieve gray or white matter infarct size or sensorimotor impairment when delivered IN, intraperitoneally, or intracerebroventricularly after term and preterm models of neonatal HI in rodents (Albertsson et al., 2014; Bonestroo et al., 2015). However, IN osteopontin-derived N134-153 peptides reduce gray matter infarct size (Albertsson et al., 2014).

#### Interferon beta

Interferon beta given IN for neonatal HI in rats penetrates the brain, reduces infarct size, increases STAT3 activation and Bcl-2 expression, and decreases cleaved caspase-3 expression (Dixon et al., 2016).

#### Glucocorticoids

Although both dexamethasone and hydrocortisone given intracerebroventricularly decrease infarct size after neonatal HI in rodents, when given IN, only males receive therapeutic benefit from the highest tested dose of hydrocortisone. Both drugs were therapeutic when HI was sensitized by lipopolysaccharide exposure (Harding et al., 2016).

#### C3a

IN C3a, after neonatal HI in mice, ameliorates reactive gliosis in the hippocampus but does not improve hippocampal tissue loss nor expression of synapsin 1 or growth-associated protein 43 (Moran et al., 2017; Pozo-Rodrigalvarez et al., 2021). The effect of IN C3a on neuronal survival and cell death is varied and may be regiospecific. Long-term memory is improved after IN C3a administration (Moran et al., 2017).

#### IRE1a inhibition

IN STF-083010, an IRE1α RNase inhibitor, reduces infarct size, neuron cell death, and cognitive and motor impairment after neonatal HI in rats (Chen et al., 2018; Huang et al., 2020). IN STF-083010 reduces TXNIP, NLRP3, and inflammatory cytokine expression with decreased gliosis (Chen et al., 2018). While IRE1α inhibition may drive many therapeutic effects after HI, the current study focuses on the inflammasome. Changes to XPB1 splicing after STF-083010 administration are not confirmed.

#### CpG-oligodeoxynucleotide

CpG-oligodeoxynucleotide reduces infarct size and cognitive and sensorimotor impairment and upregulates LC3II/I and LAMP1 expression when administered IN after neonatal HI in rodents (Ye et al., 2018a). CpG-oligodeoxynucleotide may promote autophagy and subsequent neuroprotection after HI by the toll-like receptor 9.

#### Chemerin

Human recombinant adipokine, Chemerin, reduces infarct size, decreases cleaved-caspase-3 and Bax expression, decreases neuronal cell death, and improves cognitive and motor outcomes when administered IN to rodents after neonatal HI (Zhang et al., 2019).

#### Ghrelin

IN ghrelin is brain penetrant and reduces infarct size, cognitive and sensorimotor impairment, oxidative stress, and neural cell death after neonatal HI in rodents (Huang et al., 2019).

#### Slit2

Slit2 reduces infarct size, resolves cognitive and sensorimotor impairments, and decreases neuron cell death when delivered IN after neonatal HI in rats (Kaur et al., 2019).

#### Hyaluronate-fibroblast growth factor loop peptide conjugate

IN fibroblast growth factor loop peptide conjugated to hyaluronate reduces infarct size, neural cell death, gliosis, caspase 3 expression, inflammatory cytokine expression, and sensorimotor impairment when delivered IN after neonatal HI in rodents (Kim et al., 2019).

#### Colony-stimulating factor 1

Recombinant colony-stimulating factor 1 (CSF-1) reduces infarct size and resolves cognitive and sensorimotor impairments when delivered IN after neonatal HI in rodents (Hu et al., 2020a, b). IN CSF-1 reduces inflammation and oxidative stress with a decrease in neural cell death.

#### cGAS/STING pathway inhibitors

Both cGAS inhibitor, RU.521, and upstream LINE-1 inhibitor, Stavudine, are brain penetrant, reduce infarct size, neural cell death, and cathepsin B expression when delivered IN after neonatal HI in rats (Gamdzyk et al., 2020). These findings suggest a role for the cGAS/STING signaling pathway in delayed neurodegeneration after neonatal HI.

#### Melanocortin-1 receptor signaling

IN BMS-470539, an agonist of melanocortin-1 receptor, reduces infarct size and improves cognitive and motor outcome after neonatal HI in rats (Yu et al., 2021, 2022). IN BMS-470539 increases melanocortin-1 receptor, cyclic adenosine monophosphate, phosphorylated protein kinase A, nuclear receptor-related 1, heme oxygenase-1, and Bcl-2 expression and decreases inflammatory cytokine expression. Neuron cell death and oxidative stress are decreased, and microglia shift to the M2 state (Yu et al., 2021, 2022).

#### Leukemia inhibitory factor

Leukemia inhibitory factor reduces infarct size and short-term sensorimotor impairment, improves oligodendrocyte survival and myelination, and decreases gliosis when given IN after neonatal HI in rats (Lin et al., 2020).

#### GPR39 signaling

Repeated IN dosing of TC-G1008, an activator of GPR39, after neonatal HI in rodents increases expression of sirtuin 1, peroxisome proliferator-activated receptor-gamma coactivator 1-alpha, and nuclear factor erythroid 2-related factor 2, and decreases inflammatory cytokine expression (Xie et al., 2021). IN TC-G-1008 decreases brain infarct size and improves cognitive and sensorimotor outcomes (Xie et al., 2021).

### Therapeutic impact potential

Results of the TIP are displayed in **Figure 2** for all 34 IN therapies. MSCs obtained the highest TIP score with a value of 68.0. The median TIP score was 17.0 with an interquartile range of 12.8. The highest scoring therapy overall and in the cell-based and subcellular group was MSCs with a score of 68.0. In the trophic and growth factor group, IGF-1 scored highest at 36.4, Sestrin 2 led in the cellular stress signaling group with a score of 28.0, and CSF-1 in the immunomodulatory/anti-inflammatory group with a score of 32.0.

When brain penetrance was not considered, the median TIP score was 16.0 with an interquartile range of 8.5. MSCs remained the highest scoring therapy overall and in the cell-based and subcellular group with a TIP score of 58.3. IGF-1 scored highest in the trophic and growth factor group at 31.4, Sestrin-2 scored highest in the cellular stress signaling group at 23.0, and CSF-1 scored highest in the immunomodulatory/anti-inflammatory group at 32.0.

## Conclusions

Innovative therapies for infants with HIE in developed and developing communities are urgently needed. IN therapeutic strategies pose an eminently accessible approach to HIE intervention. They are non-invasive and easily administered by both skilled and unskilled providers (Pires et al., 2009; Chung et al., 2023). Many potential IN therapeutics are less expensive than current treatment options and can permeate the CNS with a relatively low dose (Ross et al., 2004; Yang et al., 2013a). Furthermore, many IN therapy candidates may be neuroprotective when administered days after injury onset. These favorable characteristics meet serious needs in the world-wide treatment of HIE and highlight deficiencies with TH treatment, which is resource-intensive and must be initiated within hours of injury onset (Thayyil et al., 2021).

The phase I clinical trial of IN hMSCs for PAIS is relevant for HIE, as it provides precedent for the safe administration of IN therapeutics to the ischemic human neonatal brain (Baak et al., 2022). Although there is no report of a current clinical study of IN MSCs for PAIS, an additional phase 2 clinical study of cell-based therapies administered by other routes for HIE is underway. Excitingly, the first human study of an IN therapy for HIE, involving administration of own-mother’s breast milk adjunct to TH, began in early 2025 (clinicaltrials.gov ID NCT06746532). Breast milk is a complex substance containing many bioactive components, including trophic factors, cytokines, immunoglobulins, and multipotent stem cells (Ballard and Morrow, 2013). IN breast milk is therapeutically feasible in an early-phase human study of intraventricular hemorrhage in very low birthweight infants (Gallipoli et al., 2025; Sonmez Demir et al., 2025). These findings highlight the therapeutic potential of integrative therapies combining cell replacement with immunologic and trophic support. Nonetheless, clinical study of IN breast milk lacks a powered, randomized-controlled study, and no human clinical trial of any IN therapeutic for HIE has been completed. This scarcity of data highlights the continued, urgent demand for focused translational investigation. Additionally, dendrimer-bound therapeutics, now being delivered IN, were not included in this review, as they have not yet been tested for HIE (Sharma et al., 2024). The recent development of a glucose-dendrimer conjugate for seizures portends their development for HIE.

Abundant animal research is ongoing studying the IN administration of cell-based therapies for HIE (van Velthoven et al., 2010; Donega et al., 2013, 2014a, b; Ji et al., 2015; Robertson et al., 2021; Lawson et al., 2022). However, the cognitive and behavioral outcomes of such therapies in large animal models must be delineated before advancing to human study. While the study of cell-based therapies for HIE has progressed toward large animal models using other routes of administration, similar progress has not occurred with administering cell-based therapies via IN routes (Mintoft et al., 2024). Nonetheless, the therapeutic efficacy shown in existing studies administering cell-based therapies via other routes can complement the study of an IN administered cell-based therapy. Importantly, the cost, the on-site requirement for complex processing, and the storage and quality control involved with cell-based and subcellular therapies and commercial pharmaceutical support needed for widespread access to these therapies lessens and perhaps obviates their suitability as potential treatments in medically underserved communities (Barr, 2002). These concerns must be addressed before cell-based or subcellular therapies via IN or other routes emerge as a widespread clinical intervention for neonatal HIE. Fresh, own-mother’s breastmilk is feasible for neonatal intranasal administration and does not need the commercial pharmaceutical support required for other cell-based and subcellular therapies (Gallipoli et al., 2025; Sonmez Demir et al., 2025). IN breast milk may provide a feasible stem cell-containing therapy for neonates in LMICs if determined to be therapeutic for HIE in a human study. Although cell-based therapies may not meet the clinical demands of low-resourced communities, several other therapies targeting growth factor receptors, cellular stress signaling, and inflammation show promising neuroprotective capacity, including IGF-1, CSF-1, and CHF6467. While widespread therapeutic implementation presents many challenges in LMICs, we believe IN administration greatly increases the prospective feasibility of clinical implementation. Prospective feasibility of biologic administration for HIE in LMICs is supported by the therapeutic benefit of erythropoietin monotherapy in multiple clinical studies including under-resourced settings (Ivain et al., 2021).

The TIP, introduced in this review, attempts to provide a systematic method for investigators to determine where to focus efforts on the development of novel IN therapeutics. It is difficult to quantitatively compare the crude therapeutic efficacy of IN therapies due to differences in therapeutic mechanism, outcome measures, and experimental design. The TIP combines measures of therapeutic efficacy with an evaluation of evidence from individual therapies studied to provide a semi-quantitative measure of how likely therapies are to translate to a human clinical trial. Cell-based therapies demonstrate promising therapeutic potential, with MSCs and EVs having the most therapeutic evidence to date. Limitations of the TIP center primarily on the subjective assignment of weighting factors by the authors to outcome classifications. While improved neurobehavioral outcome is important for patients and families in translation of novel neurotherapeutics for HIE, brain penetrance was assigned the highest weighting factor. Neurobehavioral outcome was assigned a lower weighting factor for analysis of preclinical models because neurobehavioral outcome measures in animal models have limited translation and relevance to human. We acknowledge, however, that many therapeutics can target the brain without entering the brain parenchyma. Adjusted TIP scores with excluded brain penetrance and consequent prioritization of neurobehavioral outcome are available in the Section Therapeutic Impact Potential. Furthermore, the TIP is ignorant of differences in outcome attributable to variation in HI injury model and therapeutic dosing paradigm.

We expect IN therapeutics for HIE to rapidly advance to study in large animal models and begin human clinical trials. It is imperative that future study of IN therapies challenge therapeutic windows; consider nuances posed by animal sex, developmental timepoint, and species; include appropriate large animal models; and evaluate advanced neurobehavioral and pathological outcomes to determine functional and behavioral efficacy. IN therapies may soon play a vital role as safe, effective, and accessible treatments for HIE.

## Additional files:

***Additional Table 1:*** *Literature reviewed.*

Additional Table 1 Literature reviewed

## Data Availability

*Not applicable.*
