# Supplementary material for: Intranasal therapies for neonatal hypoxic-ischemic encephalopathy
Source: Neural Regen Res. 2025 Dec 30;21(10):4617–22. doi: 10.4103/NRR.NRR-D-25-01036 (PMC13568620; doi:10.4103/NRR.NRR-D-25-01036)
Supplement: Supplementary file 1 [file NRR-21-4617_Suppl1.pdf]

Additional Table 1: Literature reviewed

**Therapy**	**Species**	**Injury model**	**Dose**	**Dosing regimen**	**Key findings**	**Reference**
**Cell-based and subcellular**						
hCT-MSCs* (only IV) + TH	Human	HIE	1 or 2 of 2 million cells/kg	During TH then 2 mon later	MSCs are well tolerated; 5/6 babies develop low titer anti-HLA antibodies by 1 year; All babies survive with average to low-average developmental assessment at 12–17 months	Cotten et al., 2023
hMSCs	Human	PAIS	4.5-5 × 10^5 cells	Within 7 d of PAIS presentation	MSCs are well tolerated in all participants; No report of adverse events; Blood inflammation markers do not differ pre- versus post-administration; Thrombocyte levels increase but remain within normal range; 40% show abnormal corticospinal tract, and 30% exhibit abnormal motor impairment at follow-up	Baak et al., 2022
hMSCs + TH*	Piglet	HIE	2 of 3 × 10^5 cells	24- and 48-h post-HI	Expedites amplitude-integrated electroencephalogram recovery; Increases MRS phosphocreatine/inorganic phosphate; Increases OLIG2 counts; Decreases TUNEL+ cells	Robertson et al., 2021
mMSCs	Rodent	HIE (Vannucci)	5 × 10^5 cells	10 d post-HI	Improves sensorimotor function; Decreases gray and white matter area loss; MSCs respond to the ischemic brain via growth factor upregulation	van Velthoven et al., 2010

hMSCs	Rodent	HIE (Vannucci)	1 × 10^6 or 2 × 10^6 cells	10 d post-HI	2 × 10^6 IN MSCs decrease lesion volume, improve motor behavior, and reduce glial scar formation and microglial activity; huMSCs induce mNSCs to differentiate *in vitro*; CXCL10 may play an important role in migration of MSCs to the lesion site	Donega et al., 2014b
mMSCs + CXCL10	Rodent	HIE (Vannucci)	1 × 10^6 cells	10 or 17 d post-HI	Cerebral CXCL10 expression and IN MSC migration to the brain both decrease by d 17 post-HI; CXCL10 + IN MSCs at 10 and 17 d restore IN MSC migration and decrease gray matter damage; CXCL10 + IN MSCs at 17 d do not restore motor impairment but reduced glial scar and NeuN$^+$ neuron loss in perilesional area of thalamus	Hermans et al., 2024
mMSCs	Rodent	HIE (Vannucci)	1 × 10^6 cells	10 d post-HI	MSCs reach the lesion site within 2 h of IN administration; Number of MSCs at the lesion site peaks at 12 h and decreases after 72 h; Number of DCX (+), GFAP (+)/nestin (+), and NeuN (+) cells increases within 5 d of IN MSC administration leading to regeneration of somatosensory cortex and hippocampus; Resolves glial scar surrounding the HI insult	Donega et al., 2014a
mMSCs	Rodent	HIE (Vannucci)	5 × 10^7 cells	10 d post-HI	No neoplasia is observed in nasal epithelia, brain or other organs after MSC treatment; Improves sensorimotor	Donega et al., 2015

					and cognitive function up to 1 yr post-HI; Improves MAP2 and MBP positive staining at 5 wk and 14 mon post-HI	
mMSCs	Rodent	HIE (Vannucci)	2.5 × 10^7, 5 × 10^7, or 1 × 10^6 cells	3, 10, or 17 d post-HI	0.5 × 10^6 IN MSCs is the minimum effective dose with a therapeutic window of 10–17 d post-injury; MSC treatment improves behavioral and histologic outcome through 9 wk post-HI	Donega et al., 2013
Genetically modified mMSCs	Rodent	HIE (Vannucci)	5 × 10^5 cells	10 d post-HI	MSCs-BDNF improves motor outcome and decreased lesion volume compared to empty vector MSCs; MSCs-BDNF induced cell proliferation lasting at least 18 d after administration; MSCs-EGFL7 improves motor outcome but comparable lesion size to empty vector MSCs; MSCs-PSP produced comparable outcome to empty vector MSCs; MSCs-SHH worsens motor outcome and have comparable lesion volume compared to empty vector MSCs	van Velthoven et al., 2014
hMSCs	Rodent	HIE (Vannucci)	2 × 10^5 cells	24 h post-HI	Reduces total brain tissue loss; Increases hippocampal, but not somatosensory, neuron cell density; Decreases number of activated microglia and astrocytes in hippocampus but not in somatosensory cortex	McDonald et al., 2019

mMSCs + TH	Rodent	HIE (Vannucci)	1×10^6 cells	3 d post-HI	Combined MSC + TH treatment worsened cognitive-motor deficits, brain injury, and immune cell infiltration compared to each single therapy; MSCs + TH increased pro-inflammatory cytokine levels and decreased growth factor expression more than each single therapy	Herz et al., 2018
hMSCs	Rodent	Maternal LPS exposure-induced HI brain injury	1×10^6 cells	P14	Improves cortical neuron survival and laminar arrangement; Reduces motor impairment and improves exploratory behavior; Reduces astrogliosis and cytokine expression in hippocampus and cortex; Neural repair of IN hMSCs is related to PTBP-1 expression	Jiao et al., 2022
hAFS	Rodent	HIE (Vannucci)	5×10^5 cells	10 d post-HI	Restores gray and white matter area and resolves sensorimotor deficits; Reduces gliosis; Increases expression of BDNF, NGF, HGF, and SDF-1	Otani et al., 2019
hAECs	Sheep	Preterm HI	3 of $40.7 \pm 1.7 \times 10^6$	1, 3, and 10 d post-HI	Improves brain weight; Improves oligodendrocyte maturation and myelination; Reduces reactive gliosis; Attenuates cleaved-caspase-3 expression and improves neuronal survival and cortex and deep nuclei; Partially improved cortical EEG frequency distribution	van den Heuij et al., 2019
CYGB-hMSCs	Rodent	HIE (Vannucci)	1×10^6 cells	30 min post-HI	Reduces cell death greater than non-CYGB-hMSCs; Increases CYGB	Yang et al., 2020

					expression; Improves performance on Morris Water Maze and reduces p-p38/p38 ratio greater than non-CYGB-hMSCs	
hMSC EVs	Rodent	HIE (Vannucci)	10 of 3.2×10^{10} Evs	1 h post-HI then once daily for 10 d	Decreases lesion volume; Improves motor outcome; Decreases cell calcium load and increases neurite length and outgrowth *in vitro*	Turovsky et al., 2022
ciMSC EVs	Rodent	HIE (Vannuci)	3 of 1×10^5 cell equivalents	1, 3, and 5 d post-HI	ciMSC-EVs and primary MSC EVs comparably protect from HI-induced brain atrophy; ciMSC-EVs reduce gliosis, endothelial activation, and leukocyte infiltration; ciMSC-EVs downregulate pro-inflammatory cytokines in the brain and increase neural and endothelial cell proliferation, oligodendrocyte maturation, and NGF expression	Labusek et al., 2023b
ciMSC EVs + TH	Rodent	HIE (Vannuci)	3 of 1×10^5 cell equivalents	1, 3, and 5 d post-HI	ciMSC-EVs + TH, but not TH monotherapy, resolve cognitive deficits, hyperactivity, and alteration of anxiety-related behavior; ciMSC-EVs + TH reduce striatal neuron loss and decrease endothelial, microglia, and astrocyte activation and pro-inflammatory cytokine expression; ciMSC-EVs + TH induce endothelial proliferation, upregulate NGF expression, and improve oligodendrocyte maturation	Labusek et al., 2023a

WJ-MSC-EVs	Rodent	HIE + LPS sensitization	8 × 10^8 Evs/10 g	24 h post-HI	Reduce microglial activation and promotes oligodendrocyte maturation; *DROSHA* knockdown in WJ-MSCs lowered efficacy of IN EVs indicating a role for miRNAs in the therapeutic capabilities of MSC-EVs	Tscherrig et al., 2024
hNSCs	Rodent	HIE (Vannucci)	3 × 10^5 cells	24 h post-HI	Reduces upregulation of *IL-1b, p-I*κBα, and NF-κB p65; Reduces tissue loss and white matter injury; Improves cognitive and motor outcome	Ji et al., 2015
NSCs	Rodent	HIE (Vannucci)	1 × 10^6 cells	3 d post-HI	Both NSCs and NSCs overexpressing bFGF decrease gray and white matter area loss and improve sensorimotor function; NSCs overexpressing bFGF are more effective at improving somatosensory function and differentiating into neurons	Ye et al., 2018b
mNSC or brain Evs	Rodent	HIE (Vannucci)	2 of 8 × 10^9 Evs	30 min and 24 h post-HI	Reduces infarct size; NSC-EVs reduce TUNEL$^+$ cells, and Brain-EVs reduce caspase-3 expression; Brain-EVs expressed miRNAs shown to be downregulated post-HI	Lawson et al., 2022
**Trophic and growth factors**						
IGF-1	Rodent	HIE (Vannucci)	2 of 50 µg	0, 1, or 2 h post-HI	Improves neurobehavioral outcome; Inhibits neuronal apoptosis; Enhances proliferation of neuronal and oligodendroglial progenitors	Lin et al., 2009
IGF-1 + TH	Rodent	HIE (Vannucci)	2 of 50 µg	0, 2, or 4 h after TH	Hypothermia extends therapeutic window of IGF-1 from 2 to 6 h post-HI;	Lin et al., 2011

					TH + delayed IN IGF-1 decreases infiltration of polymorphonuclear leukocytes and activation of microglia/macrophages; TH + delayed IN IGF-1 attenuates NF-κB activation	
aTf	Rodent	HIE (Vannucci)	2 of 40 μg	Immediately after and 30 min after HI	Improves survival and maturation of oligodendrocytes; Improves myelination; Reduces neuron loss; Reduces astrogliosis; Increases subventricular neuronal and oligodendroglial proliferation; Reduces caspase 3 expression in corpus callosum, striatum, and cortex	Guardia Clausi et al., 2012
bFGF	Rodent	HIE (Vannucci)	Multiple of 100 ng/g	0 h after HI then twice daily for 5 d	Reduces brain infarct volume, neuron loss, and cleaved caspase 3 expression; Reduces endoplasmic reticulum stress-related protein expression	Lin et al., 2017
aFGF	Rodent	HIE (Vannucci)	15 of 100 ng/g	0 h after HI then twice daily for 1 wk	Reduces brain infarct volume, neuron loss, and cleaved caspase 3 expression; Reduces endoplasmic reticulum stress-related protein expression	Hu et al., 2017
VEGF + TH	Piglet	HIE	3 of 100 ng/kg	15 min post-HI then once daily for 3 d	Attenuates loss of cerebrovascular density in vessels/mm^2	Jain et al., 2017
CHF6467	Rodent	HIE (Vannucci)	4 or 20 μg/kg	10 min, 1 h, or 3 h post-HI	Reduces infarct volume; Improves motor and learning and memory deficits; Reduces mRNA upregulation of neuroinflammatory cytokines; Decreases plasma NF-L; Reduces	Landucci et al., 2024

					neuronal death and neurotransmission impairment in hippocampal slices exposed to oxygen and glucose deprivation	
BDNF + TH	Rodent	HIE (Vannucci)	7 of 15 µg	Once daily for 7 d starting 24 h post-HI	Improves performance on a novel object recognition task; Increases synaptophysin and MAP2 expression in hippocampus and neocortex	Sims et al., 2024
**Cellular stress signaling**						
PAI-1	Rodent	HIE and HIE + LPS Sensitization	2.88 µg	30 or 120 min post-HI	Reduces total infarct area, plasminogen activation, NF-κB signaling, and markers of cerebral inflammation	Yang et al., 2013b
Sestrin2	Rodent	HIE (Vannucci)	0.3, 1.0, or 3.0 µg	1 h post-HI	Activates the AMP-activated protein kinase pathway; Reduces infarct area, neuronal apoptosis, and ventricular area enlargement; Improves neurobehavioral outcome	Shi et al., 2017
Sestrin2	Rodent	HIE (Vannucci)	0.3, 1.0, or 3.0 µg	1 h post-HI	Reduces infarct size and cerebral edema; Improves performance on righting and geotaxis reflexes; Reduces VEGF activation; Improves junction protein expression; Stabilizes BBB permeability	Shi et al., 2016
miR-210-LNA	Rodent	HIE (Vannucci)	100 or 200 pmol	4 h post-HI	Reduces infarct size and sensorimotor deficits	Ma et al., 2016
Adiponectin	Rodent	HIE (Vannucci)	0.05, 0.1, or 0.3 mg/kg	1 h post-HI	Reduces infarct size and sensorimotor and cognitive disfunction; Upregulates AdipR1 and APPL1 expression in	Xu et al., 2018

					neurons; Upregulates AMPK phosphorylation	
GW0742	Rodent	HIE (Vannucci)	2 of 25, 100, or 400 μg/kg	1 and 24 h post-HI	Reduces infarct size, neuronal death, and cognitive and sensorimotor disfunction; Upregulates nuclear PPAR-b/d; Reduces TXNIP levels and inhibits p-ASK1/p-p38 signaling; Increases miR-17-5p expression	Gamdzyk et al., 2018
HIF stabilization**,***; GSK360A	Rodent	HIE (Vannucci)	30–60 μg/dag (IN), 12 μg/dag (ICV), or 100–500 μg/dag (IP)	Within 1 h of HI	IN and ICV routes increase erythropoietin, heme oxygenase 1, and glucose transporter 1 transcripts; Promotes neuronal and oligodendroglial survival; Fails to relieve cerebral infarct size	Kuan et al., 2021
Insulin	Rodent	HIE (Vannucci)	50 μg	Immediately after HI	Reduces sensorimotor deficits; Reduces infarct size; Reduces neuron cell death and cleaved-caspase-3 expression; Increases Akt phosphorylation	Talati et al., 2024
Cerium vanadate nanoenzyme	Rodent	HIE (Vannucci)	0.192, 0.384, 0.480, 0.960, or 4.8 μg	24 and 47 h post-HI	Reduces infarct size, neuron loss, and cleaved caspase 3 expression; Reduces reactive oxygen species and improves Nrf2 expression; Reduces gliosis and inflammatory cytokine expression; Improves dendritic spine density; Relieves sensorimotor and cognitive deficits	Jiang et al., 2024
**Immunomodulatory and anti-inflammatory**						
Anti-NF-κB peptides	Rodent	HIE + LPS sensitization	1.4 mg/kg	10 min post-HI	Attenuates NF-κB signaling, microglial activation, and cortical infarct size when	Yang et al., 2013a

					HI injury is comorbid with lipopolysaccharide sensitization	
Osteopontin***	Rodent	Preterm HIE	2 of 0.6 μg	Immediately before and after hypoxia	Fails to resolve gray or white matter area loss with IN or ICV administration; Osteopontin-derived N134-153 and C154-198 peptides decrease gray matter area loss	Albertsson et al., 2014
TAT-osteopontin peptide	Rodent	HIE (Vannucci)	350 or 2100 ng	Immediately post-HI then at 3 h, 1, 2, and 3 d post-HI	Failed to resolve gray or white matter area loss by any route; Did not improve sensorimotor outcome	Bonestroo et al., 2015
IFN-β**	Rodent	HIE (Vannucci)	3 of 0.03, 0.3, or 1.0 μg/kg	2 h post-HI then once daily for 3 d	Reduces infarct volume; Improves short-term neurobehavioral outcome; Shows no effect on cerebral edema; Stimulates Stat3 and Bcl-2 expression leading to a decrease in cleaved caspase-3 expression; There were no difference in efficacy between IN and IP routes	Dixon et al., 2016
Glucocorticoids***	Rodent	HIE and HIE + LPS sensitization	IN: 1, 3, or 30 μg dexamethasone; 50, 100, or 300 μg hydrocortisone	2 h post-HI	Only ICV dexamethasone reduces cortical infarct size; ICV and IN hydrocortisone reduces cortical infarct size (in males only with IN); Both ICV and IN hydrocortisone reduce cortical infarct size in both sexes after lipopolysaccharide sensitization	Harding et al., 2016
C3a	Rodent	HIE (Vannucci)	3 of 2.56 μg/kg	1 h post-HI then once daily for 3 d	Improves performance in an object recognition task; Ameliorates reactive gliosis in the hippocampus; Does not affect tissue loss, neuronal cell density,	Moran et al., 2017

					expression of synapsin I, or expression of growth associated protein 43	
C3a	Rodent	HIE (Vannucci)	3 of 2.56 μg/kg	1 h post-HI then once daily for 3 d	Reduces neuron cell death and astrogliosis in hippocampus	Pozo-Rodrigalvarez et al., 2021
IRE1a inhibition; STF-083010	Rodent	HIE (Vannucci)	3 of 15 μg or 1 of 45 μg	1, 24, and 48 h post-HI or 1 h post-HI	Improves neurobehavioral outcome; Attenuates decrease in miR-17-5p expression; Reduces TXNIP expression, NLRP3 inflammasome activation, caspase-1 cleavage, IL-1β production, and infarct size volume; IP administration was not therapeutic and increased mortality	Chen et al., 2018
IRE1a inhibition; STF-083010	Rodent	HIE (Vannucci)	45 μg	1 h post-HI	Reduces infarct size, neuron cell death, and short-term sensorimotor impairment; Increases miR-125-2-3p and inflammatory cytokine expression; These effects took place through the miR-125/NLRP1 pathway	Huang et al., 2020
CpG-ODN	Rodent	HIE (Vannucci)	0.35, 0.70, or 1.40 mg/kg	1 h post-HI	Reduces infarct size and cognitive and sensorimotor impairment; Upregulates LAMP1 and LC3I/II; These effects take place through the TLR9/pAMPK signaling pathway	Ye et al., 2018a
Chemerin	Rodent	HIE (Vannucci)	3, 9 or 27 μg/kg/day	1 h post-HI then once daily for 3 or 7 d	Reduces infarct size and resolves cognitive and motor impairment; Decreases cleaved caspase 3 and bax expression; Decreases neuronal cell death; Decreases neuronal oxidative	Zhang et al., 2019

					stress and lipid peroxidation; IN Chemerin suppresses apoptosis through the chemR23/CAMKK2/AMPK signaling pathway	
Ghrelin	Rodent	HIE (Vannucci)	2 of 0.02, 0.04, or 0.12 μg/kg	1 and 24 h post-HI	Reduces infarct size and cognitive and sensorimotor impairment; GHSR-1a colocalizes with neurons; Reduces oxidative stress and neural cell death; These effects took place through the GHSR-1a/AMPK/Sirt1/PGC-1a/UCP2 signaling pathway	Huang et al., 2019
Slit2	Rodent	HIE (Vannucci)	3, 10, or 30 μg	1 h post-HI	Reduces infarct size and cognitive and sensorimotor impairment; Reduces neuron cell death; These effects take place through the Slit2/Robo1/srGAP1/RhoA signaling pathway	Kaur et al., 2019
Hyaluronate-FG loop peptide	Rodent	HIE (Vannucci)	10 kDa HA, FGL, 10 kDa HA–FGLala conjugate, and 10 kDa HA–FGL conjugate	90 min post-HI	Decreases infarct size, neural cell death, and macrophage proliferation; Reduces astrogliosis, caspase 3 upregulation, and inflammatory cytokine expression; Resolves sensorimotor impairment	Kim et al., 2019
CSF-1	Rodent	HIE (Vannucci)	40, 80, or 160 μg	1 h post-HI	Reduces infarct size and cognitive and sensorimotor impairment; CSF-1 and its receptor colocalize with microglia; Reduces inflammatory cytokine expression; These effects take place through the	Hu et al., 2020b

					CSF1R/PLCG2/PKCe/CREB signaling pathway	
CSF-1	Rodent	HIE (Vannucci)	2 of 80 µg/kg	1 and 24 h post-HI	Reduces infarct size and cognitive and sensorimotor impairment; CSF-1 and its receptor colocalize with some neurons and astrocytes; Reduces oxidative stress and neuronal cell death; These effects take place through the CSF1R/PLCG2/PKA/UCP2 signaling pathway	Hu et al., 2020a
cGAS inhibitor, RU.521, or LINE-1 inhibitor, stavudine	Rodent	HIE (Vannucci)	3 of 4.5, 45, or 450 µg/kg (RU.521) or 2 of 500 µg/kg (Stavudine)	2, 24, and 48 h post-HI (RU.521) or 2 and 24 h post-HI (Stavudine)	RU.521 reduces infarct size, cathepsin B expression, and neural cell death; Therapeutic effects of RU.521 are blocked by STING activation; Stavudine reduces infarct size, cathepsin B expression and neural cell death	Gamdzyk et al., 2020
MC1R activation; BMS-470539	Rodent	HIE (Vannucci)	50, 160, or 500 µg/kg	1 h post-HI	Reduces percent infarct area, brain atrophy, and inflammatory markers; Ameliorates short- and long-term cognitive deficits; Increases expression of CD206, MC1R, cAMP, p-PKA, and Nurr1	Yu et al., 2021
MC1R activation; BMS-470539	Rodent	HIE (Vannucci)	160 µg/kg	1 h post-HI	Attenuates infarct size and short-term neurologic deficit; Reduces cleaved caspase 3; Upregulates expression of MC1R, cAMP, p-PKA, Nurr1, HO-1, and Bcl-2 and downregulates expression	Yu et al., 2022

					of 4-HNE and Bax; Reduces neuron cell death and oxidative stress	
LIF	Rodent	HIE (Vannucci)	6 of 0.02 μg	3 d post-HI then twice daily for 3 d	Increases pStat-3 expression; Decreases gliosis; Improves oligodendrocyte survival and myelination; Reduces infarct size and resolves sensorimotor impairment	Lin et al., 2020
GPR39 activation; TC-G1008	Rodent	HIE (Vannucci)	4 or 1 of 5, 15, or 45 mg/kg	1, 25, 49, and 73 or 1 h post-HI	Reduces percent infarcted area; Improves short-term and long-term cognitive deficits; Increases expression of SIRT1, PGC-1α and Nrf2; Downregulates expression of IL-6, IL-1β, and TNF-α	Xie et al., 2021

*Study also assessed IV administration of the therapeutic, **Study also assessed IP administration of the therapeutic, ***Study also assessed ICV injection of the therapeutic. AMPK: AMP-activated protein kinase; BDNF: brain-derived neurotrophic factor; CXCL10: C-X-C motif chemokine ligand 10; DCX: doublecortin; EGFL7: epidermal growth factor-like domain 7; EEG: electroencephalography; EV: extracellular vesicle; GFAP: glial fibrillary acidic protein; HI: hypoxia-ischemia; HIE: hypoxic-ischemic encephalopathy; h: human; HLA: human leukocyte antigen; ICV: intracerebroventricular; IGF-1: insulin-like growth factor 1; IL-6: interleukin-6; IN: intranasal; IP: intraperitoneal; IV: intravenous; LPS: lipopolysaccharide; MAP2: microtubule-associated protein 2; MBP: myelin basic protein; MRS: magnetic resonance spectroscopy; MSC: mesenchymal stromal cell; NF-κB: nuclear factor kappa B; OLIG2: oligodendrocyte transcription factor 2; P: postnatal day; PAIS: perinatal arterial ischemic stroke; PPAR-β/δ: peroxisome proliferator-activated receptor beta/delta; PSP: phosphoserine phosphatase; PTBP-1: polypyrimidine tract binding protein 1; ROS: reactive oxygen species; SHH: sonic hedgehog; TH: therapeutic hypothermia; TUNEL: terminal deoxynucleotidyl transferase dUTP nick end labeling; VEGF: vascular endothelial growth factor
